# Prevalence of Vancomycin resistant *enterococci* (VRE) in Ethiopia: a systematic review and meta-analysis

**DOI:** 10.1186/s12879-020-4833-2

**Published:** 2020-02-11

**Authors:** Addisu Melese, Chalachew Genet, Tesfaye Andualem

**Affiliations:** 1 Department of Medical Laboratory Science, College of Medicine and Health Sciences, Bahir Dar University, Bahir Dar, Ethiopia; 2 Department of Medical Laboratory Science, College of Health Sciences, Debre Tabor University, Debre Tabor, Ethiopia

**Keywords:** Enterococcus, Vancomycin resistance, Systematic review, Ethiopia

## Abstract

**Background:**

The emergence of Vancomycin resistant *enterococci* (VRE) poses a major public health problem since it was first reported. Although the rising rates of VRE infections are being reported elsewhere in the worldwide; there is limited national pooled data in Ethiopia. Therefore, this study was aimed to estimate the pooled prevalence of VRE and antimicrobial resistance profiles of *enterococci* in Ethiopia.

**Methods:**

Literature search was done at PubMed, EMBASE, Google scholar, African Journals online (AJOL) and Addis Ababa University repository following the Preferred Reporting Items for Systematic Reviews and Meta-Analyses (PRISMA) guideline. Both published and unpublished studies reporting the prevalence of VRE until June 30, 2019 were included. Data were extracted using Microsoft Excel and copied to Comprehensive Meta-analysis (CMA 2.0) for analysis. Pooled estimate of VRE was computed using the random effects model and the 95% CIs. The level of heterogeneity was assessed using Cochran’s Q and I^2^ tests. Publication bias was checked by visual inspection of funnel plots and Begg’s and/or Egger’s test.

**Results:**

Twenty studies fulfilled the eligibility criteria and found with relevant data. A total of 831 *enterococci* and 71 VRE isolates were included in the analysis. The pooled prevalence of VRE was 14.8% (95% CI; 8.7–24.3; *I*^*2*^ = 74.05%; *P* <  0.001). Compared to vancomycin resistance, *enterococci* had higher rate of resistance to Penicillin (60.7%), Amoxicillin (56.5%), Doxycycline (55.1%) and Tetracycline (53.7%). Relatively low rate of resistance was found for Daptomycin and Linezolid with a pooled estimate of 3.2% (95% CI, 0.5–19.7%) and 9.9% (95% CI, 2.8–29.0%); respectively. The overall pooled multidrug resistance (MDR) rate of *enterococci* was 60.0% (95% CI, 42.9–75.0%).

**Conclusion:**

The prevalence of VRE and drug resistant *enterococci* are on the rise in Ethiopia. Enterococcal isolates showed resistance to one or more of the commonly prescribed drugs in different or the same drug lines. Multidrug resistant (MDR) *enterococci* were also found. Although the rates were low, the emergence of resistance to Daptomycin and Linezolid is an alarm for searching new ways for the treatment and control of VRE infections. Adherence to antimicrobial stewardship, comprehensive testing and ongoing monitoring of VRE infections in the health care settings are required.

## Background

Today, antimicrobial resistance (AMR) is one of the most important public health problem in the world and continues to challenge treatment especially in bacteria [1]. Widespread use and misuse of antibiotics is thought to increase the prevalence and emergence of resistance bacterial strains. As a growing problem; AMR complicates the treatment of bacterial infections leading to increased mortality, morbidity and healthcare related costs. The emergence of Vancomycin resistant *enterococci* (VRE) poses a major public health problem since it was first reported. VRE are among the most common resistant pathogens frequently causing healthcare associated infections and a growing concern for health care professionals [1–4].

*Enterococci* are gram-positive bacterial flora of the intestinal tract of humans, animals and birds [5–7]. Despite their commensal characteristics, they cause serious nosocomial infections in humans including urinary tract, bloodstream infections and endocarditis [8]. They are “tough bugs” that can survive in/and on the environment for long periods and became one of the main nosocomial pathogens. *Enterococci* are also able to form biofilms that contribute to the virulence, resistance to antibiotics and phagocytosis making their eradication extremely difficult [9, 10].

*Enterococci* become resistant to a variety of antimicrobials through intrinsic and acquired mechanisms. Isolates of *E. gallinarum* and *E. flavescens* develop an inherent, low-level resistance to Vancomycin [11]. *Enterococci* readily accumulate mutations and exogenous genes that confer additional resistance. They develop resistance to vancomycin by exchange of genetic material among themselves and/or with another genera [12]. The *enterococci* may acquire resistance through *van* associated genetic elements (*vanA, vanB, vanD, vanE, vanG, vanL*); of which *vanA* and *vanB* are the most prevalent genotypes in clinical isolates [11, 13, 14]. The *vanA* and *vanB* gene clusters are most commonly found in *E. faecium* and increasingly reported throughout the world [12, 15]. Other transposable elements are also reported to be involved in the spread of antimicrobial resistance [16].

Vancomycin was considered as one of the last lines of treatment against multidrug resistant organisms including ampicillin resistant *enterococci* and methicillin-resistant *Staphylococcus aureus* (MRSA) [8]. However, *enterococci* develop high level of resistance and the incidences of VRE infections among hospitalized patients has increased rapidly [9, 13, 17]. Infections due to VRE have been also reported to be associated with longer hospital stays, increased mortality and higher healthcare costs than infections with vancomycin susceptible *enterococci* [15, 18–20].

Enterococcal infections are now getting attention due to their ability to develop resistance to multiple antimicrobial agents which probably explain their large part of isolation in nosocomial infections [21, 22]. The two species (*E. faecalis* and *E. faecium)* are responsible for majority of the infections in humans. They are also constituting a reservoir for antibiotic resistance among the gut enterococci [23]. In 2017, the World Health Organization (WHO) has published the priority lists of antibiotic-resistant bacteria to guide research, discovery, and development of new antibiotics. Hence; Vancomycin resistant *E. faecium* was categorized as high priority pathogens for which new and effective treatments are need [24]. Reports are also emerging on the development of resistance to Daptomycin and Linezolid which are being used to treat Vancomycin resistant enterococcal infections [14]; this could explain the challenging nature of these bacteria in the current medicine and as well as to the future. Other studies reported the continuous increase of VRE causing nosocomial infections [25].

In Ethiopia; different reports showed that antimicrobials are widely misused by health care providers, unskilled practitioners, animal husbandry operators and drug users. Antimicrobial misuse is one of the major driver and contributor of the emergence and survival of resistance strains. To prevent and contain the spread of drug resistance, the Ethiopian Public Health Institute (EPHI) established AMR surveillance centers and identified national priority surveillance pathogens in 2017 [4]. A previous systematic review has also reported the growing challenges of antibacterial drug resistance in Ethiopia [26]; but VRE were included neither in the national priority surveillance pathogens nor in previous systematic reviews. Although the rising rates of VRE infections are being reported elsewhere in the worldwide; there is limited national pooled data in Ethiopia. Therefore; this study was aimed at summarizing the findings of local studies to estimate the pooled prevalence of VRE and antimicrobial resistance profiles of *enterococci* in Ethiopia.

## Methods

### Search strategy

**Fig. 1:**
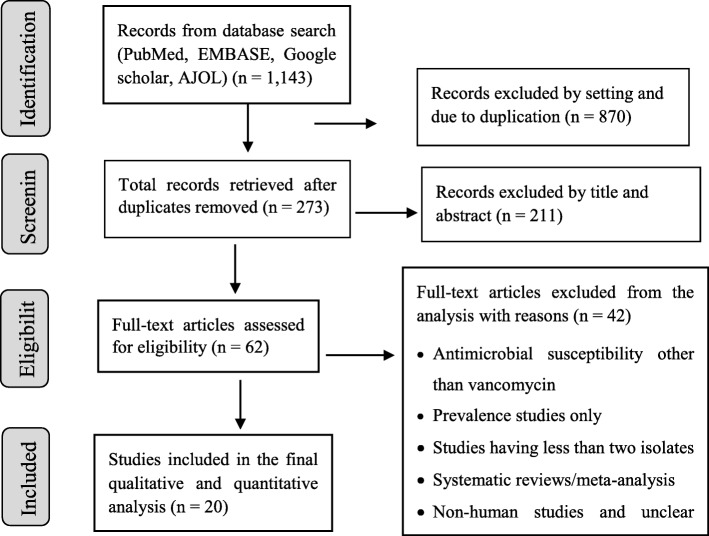
PRISMA flow chart of study selection

A comprehensive search was conducted at PubMed, EMBASE, Google scholar and African journals online (AJOL). To include unpublished studies (theses, dissertations); the repository of Addis Ababa University was searched. Reference lists of included studies were also sought. The database search was done following the PRISMA guideline/checklists [27] (Fig. 1). The PubMed was searched using MeSH terms and Boolean operators. The search string in PubMed was: ((((((((Enteroccoc*) OR *Enterococcus faecalis*) OR *Enterococcus faecium*) OR *E. faecalis* OR *E. faecium* AND Vancomycin resistan*) OR antibiotic resistan*) OR antimicrobial resistan*) OR drug resistan*) OR VRE) AND Ethiopia)))))))). Search results were combined in to EndNote X6 (Clarivate Analytics USA) and duplicates were removed. Studies published/reported up to June 30, 2019 and fulfilled the eligibility criteria (Table 1) were included.

**Table 1:** Eligibility criteria

Inclusion criteria	Exclusion criteria
• Study settings: conducted in Ethiopian on any settings • Study subjects/population: humans • Study design: any study reported the prevalence of VRE or numbers of VRE and total enterococci isolates • Sample size: studies isolated not less than two enterococci • Language: published/reported in English • Type of study: peer-reviewed, full text available before June 30, 2019	• Studies on antimicrobial susceptibility tests other than vancomycin (studies that did not include VRE) • Prevalence studies only • Studies having less than two isolates • Studies not reporting enterococcal isolates separately (no population denominator) • Reviews, comments and duplications • Studies on non-human subjects

### Quality assessment

The quality of included studies was assessed by the Joanna Briggs Institute (JBI) critical appraisal checklist for prevalence data [28] (additional file 1); which contains nine sections. The assessment was done independently by two authors (AM and TA). Studies were included in the analysis if consensus was reached among the two reviewers. The quality of the 20 included studies is given in (additional file 2).

### Data extraction

After studies were identified based on the predefined eligibility criteria; author name with year of publication, study period, region of study, study design, sample size, study population, types of specimens, antimicrobial susceptibility testing (AST) methods, number of isolates (both the total and vancomycin resistant *enterococci*), types of isolated species and history of publication were extracted using Microsoft Excel 2013 data collection sheet especially designed for this study. Resistance profiles of *enterococci* to other antimicrobials were also extracted and the study level proportions were pooled. The data extraction was done independently by two authors (AM and TA).

### Data analysis

Whenever studies were not reporting the prevalence of VRE, it was calculated by dividing the numbers of VRE isolates to the total numbers of tested enterococcal isolates and multiplying by 100. Studies reporting a zero number of VRE isolates were imputed to 0.5 as a continuity correction to be include in the meta-analysis [29]. Subgroup analyses were done by the study region, study period, publication history, AST and types of specimens used to isolate *enterococci*.

Acknowledging the presence of heterogeneity in observational studies conducted in diverse settings, the random effects model was used in determining the pooled prevalence of VRE as well as resistance to other antimicrobials. Heterogeneity was evaluated by the Cochran’s Q-test and I^2^ statistics. Funnel plots were drawn to see the presence of publication bias and the Begg’s rank correlation and Egger’s regression tests were used to quantify the degree of publication bias. *P-values* <  0.05 in any of the Begg’s rank correlation and Egger’s regression tests were indicative of significant publication bias. In asymmetrical funnel plots, the Trim-and-Fill method was applied to include missing studies and estimate adjusted effect sizes. Sensitivity analysis in a leave-one-out approach was done to see the stability of the pooled prevalence of VRE and to explore the potential source of heterogeneity between studies. Data were analyzed using CMA version 2.0 for windows and used to generate forest and funnel plots.

## Results

### Study selection

The results of database search and process of study selection is shown in the flow chart below (Fig. 1). The search returned 1143 records; of which 62 studies were subjected for full text review for inclusion against the eligibility criteria. Finally, 42 studies were excluded and only 20 were included in our analysis.

### Characteristics of included studies

All of the 20 studies included in this review were cross-sectional by design. Most of the studies were reported from Amhara region (*n* = 8) [30–37] and Addis Ababa (*n* = 7) [38–44]. The remaining studies were from Oromia (*n* = 4) [45–48] and Southern nations (*n* = 1) [23]. Studies were not available from administrative regions of Tigray, Afar, Dire Dawa, Harari, Somali, Gambela and Benishangul-Gumuz. Nineteen studies were conducted in hospital settings. Among the 6017 study participants included, 831 *enterococci* were isolated and tested with a variety of antimicrobials; of which 71 isolates were resistant to vancomycin. Stool, urine, blood and swab specimens were used to isolate *enterococci*. The highest numbers of enterococcal and VRE isolates were identified from stool followed by multi-site specimens.

Seventeen studies used disc diffusion and three studies employed dilution/minimum inhibitory concentration (MIC) as antimicrobial susceptibility testing (AST) method to determine Vancomycin resistance. Resistance to antimicrobial agents by either methods was defined based on the performance standards for antimicrobial susceptibility testing guidelines prepared by Clinical and Laboratory Standards Institute (CLSI, various editions). The prevalence of VRE ranged from 1.8% in Jimma to 60% in Addis Ababa. Species level *enterococci* were reported by four studies [23, 39, 47, 48] and *E. faecalis* and *E. faecium* were the most frequently isolated species. Six of the included studies were unpublished and 14 were published between 2013 and 2019. Details of the characteristics of the included studies is summarized in (Table 2) below.

**Table 2:** Lists and characteristics of included studies

Author, publication year	Study period	Study area/ region	Study design	Study subjects	Sample size	Prevalence of enterococci, N (%)	Type of specimen	AST method	Prevalence of VRE, N (%)	Types of isolates (species)	Publication history
Abamecha, 2015 [48]	January to July 2013	Jima University Specialized Hospital, Oromia	CS	Hospitalized patients	150	114 (76.00)	Stool, rectal swabs	Disc diffusion, MIC for VRE	2 (1.8)	*E.faecium, E.faecalis, E.gallinarum, E.casseliflavus, E.durans*	*Published*
Abebe, 2014 [37]	July to September 2013	University of Gondar Teaching Hospital, Amhara	CS	HIV positive and HIV negative clients	226	201 (88.94)	Stool	Disc diffusion	11 (5.5)	Not identified to species level	*Published*
Agegne, 2018 [36]	February to May 2017	West Amhara Hospitals, Amhara	CS	HIV patients on ART	349	220 (63.04)	Stool	Disc diffusion	17 (7.7)	Not identified to species level	*Published*
Ali, 2018 [35]	February to May, 2017	Dessie Referral Hospital, Amhara	CS	HIV positive and HIV negative clients	300	112 (37.33)	Stool	Disc diffusion	7 (6.3)	Not identified to species level	*Published*
Ayelign, 2018 [33]	February to June 2015	University of Gondar Hospital, Amhara	CS	Pediatric patients	310	3 (0.97)	Urine	Disc diffusion	1 (33.3)	Not identified to species level	*Published*
Birri, 2013 [23]	Not reported	Dilla town, SNNPR	CS	Healthy infants aged 3 to 26 weeks	28	53 (189.29)^a^	Stool	Dilution/MIC	1 (1.9)	*E.faecium, E.faecalis, E.avium, E.canintestini, E.maldoratus, E.raffinosus, E.gallinarum*	*Published*
Eshetu, 2017 [44]	April to September 2016	Tikur Anbessa Specialized Hospital, Addis Ababa	CS	Blood stream infection suspects	422	5 (1.18)	Blood	Disc diffusion	3 (60.0)	Not identified to species level	*Unpublished*
Fentie, 2018 [32]	February to April 2017	University of Gondar teaching Hospital, Amhara	CS	Cancer patients	216	2 (0.93)	Blood, urine, wound swab, ear discharge	Disc diffusion	1 (50.0)	Not identified to species level	*Published*
Ferede, 2018 [41]	April to May 2016	Black Lion/Tikur Anbesa Specialized Hospital, Addis Ababa	CS	Patients suspected for UTI, wound infection, septicemia, endocarditis, meningitis	422	15 (3.55)	Blood, urine, body fluid, CSF, Pus	Disc diffusion	1 (6.7)	Not identified to species level	*Published*
Gebrish, 2019 [47]	February to March 2016	Jimma University Specialized Hospital, Oromia	CS	Hospitalized pediatric patients	52	12 (23.08)	Stool, rectal swabs	Disc diffusion	1 (8.3)	*E.faecalis, E.faecalis, E.gallinarum*	*Published*
Jemal, 2017 [34]	July to December 2016	Felege Hiwot Referral Hospital, Amhara	CS	HIV patients on ART	384	4 (1.04)	Blood	Disc diffusion	0.5^b^ (10.0)	Not identified to species level	*Unpublished*
Lega, 2015 [42]	April to July 2015	Yekatit 12 Hospital Medical College, Addis Ababa	CS	Diabetic patients	246	2 (0.81)	Urine	Disc diffusion	1 (50.0)	Not identified to species level	*Unpublished*
Mitiku, 2018 [40]	September 2017 to June 2018	Tikur Anbesa Specialized Hospital, Addis Ababa	CS	Under 5 children with febrile illness	340	11 (3.24)	Blood	Disc diffusion	0.5^b^ (4.2)	Not identified to species level	*Unpublished*
Mohammed, 2017 [31]	March to May, 2014	University of Gondar Referral Hospital, Amhara	CS	Patients with wound infections	137	2 (1.46)	Wound swab	Disc diffusion	1 (50.0)	Not identified to species level	*Published*
Molalign, 2016 [39]	September 2015 to May 2016	Arsho Advanced Medical laboratory, Addis Ababa	CS	UTI patients	712	15 (2.11)	Urine	Dilution/MIC	7 (46.7)	*E.faecalis,* *E.gallinarum*	*Unpublished*
Sorsa, 2019 [46]	April 2016 to May 2017	Asella teaching and referral hospital, Oromia	CS	Neonates with sepsis	303	6 (1.98)	Blood	Disc diffusion	1 (16.7)	Not identified to species level	*Published*
Teklehaymanot, 2016	July to September 2015	Tikur Anbessa Specialized Hospital, Addis Ababa	CS	Patients suspected for body fluid pathogens	384	2 (0.52)	CSF, ascites, pleural fluid, synovial fluid	Disc diffusion	0.5 ^b^ (16.7)	Not identified to species level	*Unpublished*
Toru, 2018 [45]	April to September 2016	Jimma University Specialized Hospital, Oromia	CS	Pediatric patients (< 15 years)	403	22 (5.46)	Urine, blood, swabs, closed abscess, body fluids, CSF	Disc diffusion	5 (22.7)	Not identified to species level	*Published*
Woldemariam, 2019 [38]	April to July 2015	St. Paul Specialized Hospital Millennium Medical College, Addis Ababa	CS	Adult diabetic patients	248	6 (2.42)	Urine	Disc diffusion	1 (16.7)	Not identified to species level	*Published*
Yilema, 2017 [30]	February to May 2014	University of Gondar Teaching Hospital, Amhara	CS	Patients requiring culture and AST	385	24 (6.23)	Urine, blood, wound swabs, ear discharge, ascites, abscess	Disc diffusion	10 (41.7)	Not identified to species level	*Published*

*AST* Antimicrobial Susceptibility testing, *ART* Antiretroviral Therapy, *CS* Cross-sectional, *CSF* Cerebrospinal Fluid, *VRE* Vancomycin resistant enterococci, *MIC* Minimum Inhibitory Concentration, *SNNPR* Southern Nations, Nationalities and Peoples Region

^a^: multiple enterococcal species were isolated from a single infant; ^b^: 0.5 was added as a continuity correction to include the study in the analysis

### Pooled prevalence of VRE

**Fig. 2:**
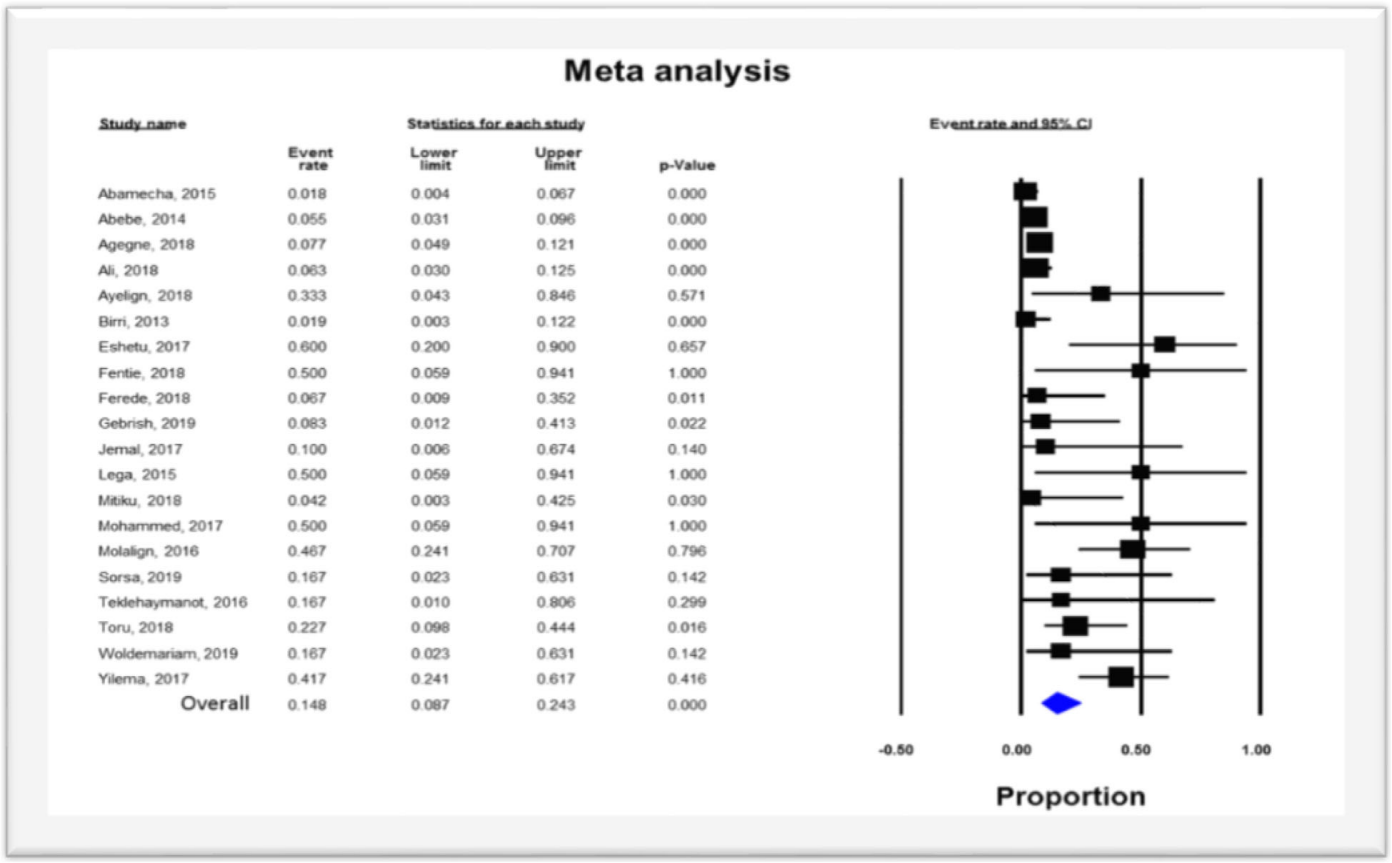
Forest plot showing the pooled prevalence of VRE in Ethiopia

**Fig. 3:**
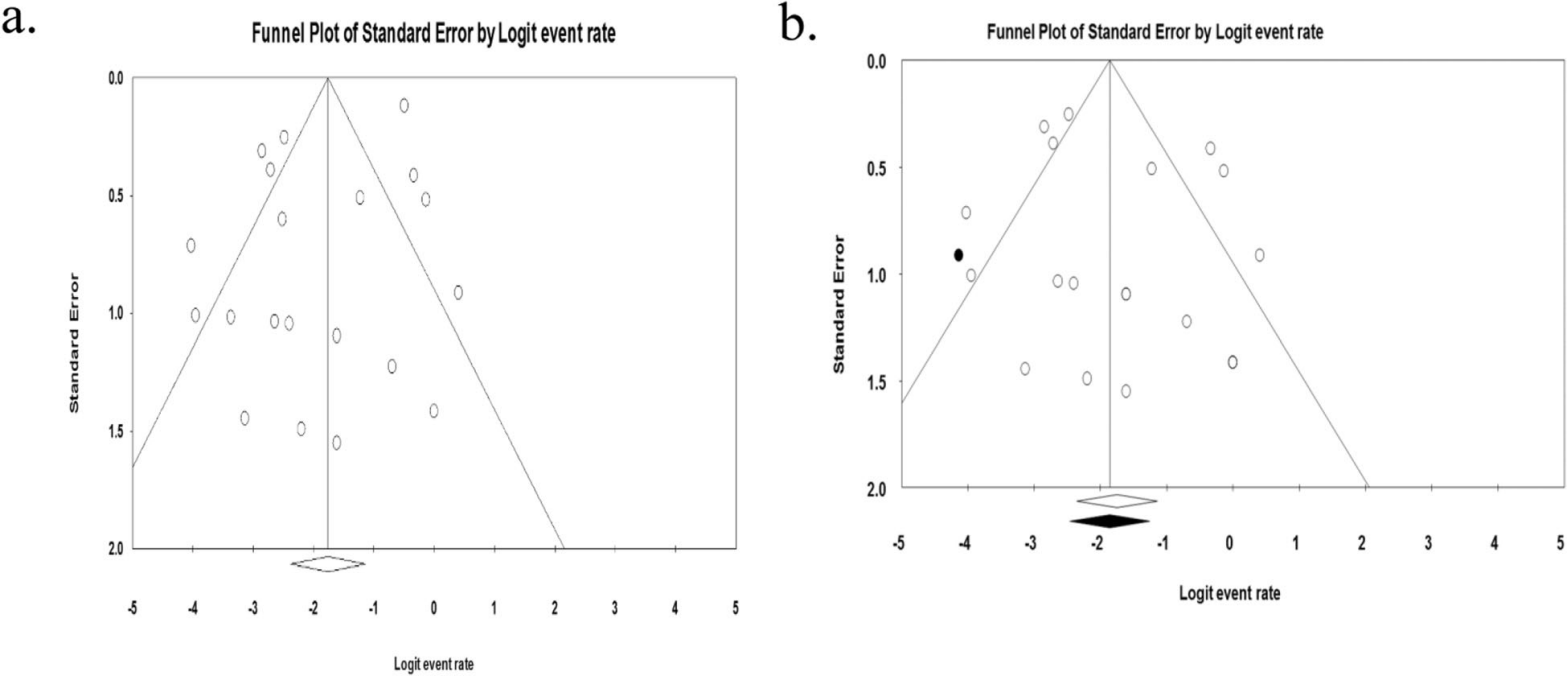
Funnel plot showing publication bias; before (**a**) and after (**b**) the Trim-and-Fill method is applied

The pooled prevalence of VRE was estimated at 14.8% (95% CI; 8.7–24.3%; I^2^ = 74.05%; *P* <  0.001) (Fig. 2). Significant heterogeneity (*Q* = 73.21; *I*^*2*^ = 74.05%; *P* <  0.001) was observed in the estimation of overall pooled result. But, the sensitivity analysis revealed that no single study significantly influenced the heterogeneity and pooled prevalence of VRE. The pooled prevalence of VRE in the sensitivity analysis ranged from 13.2 to 16.7% which lies within the 95% CI bounds of the overall pooled estimate. The presence of publication bias was observed from the drawn asymmetric funnel plot (Fig. 3a). The Trim-and-Fill method was then applied to include the “missing” studies from the analysis. The asymmetric studies were trimmed to locate the unbiased effect and fills the plot by re-inserting the trimmed studies as well as their imputed counterparts. Accordingly, one study was missed and fall at the left side of the pooled estimate (Fig. 3b). In the Trim-and-Fill method, the adjusted estimate of VRE was 13.5% (95% CI; 7.8–22.2%); almost similar with the original pooled estimate. The Egger’s regression (intercept = 0.91; 95% CI; − 0.75 – 2.57; *p* = 0.263) and Begg’s rank test (*p* = 0.381) did not suggest significant publication bias.

### Subgroup prevalence of VRE

**Fig. 4:**
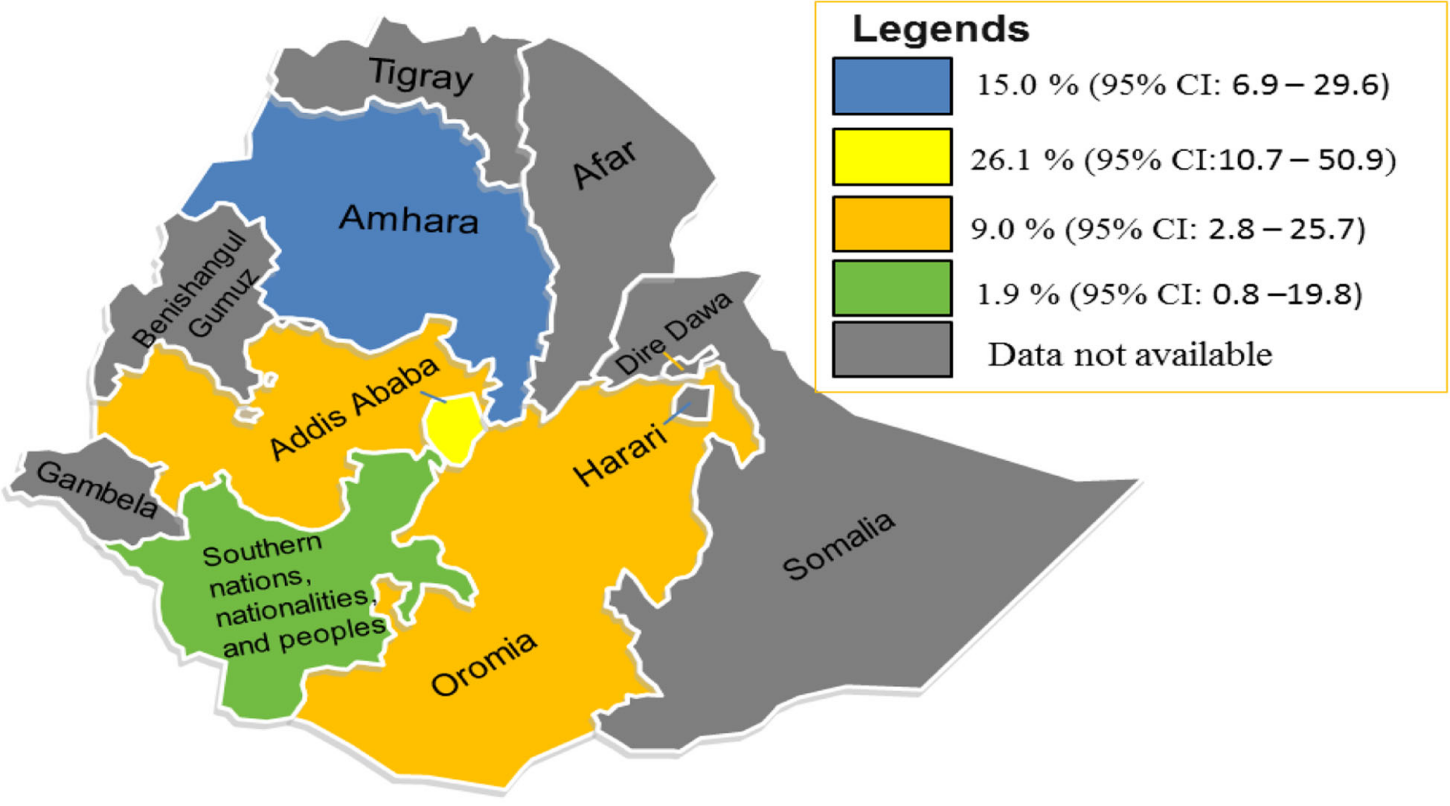
Map showing regional distribution of VRE in Ethiopia; *Map adapted from en.Wikipedia.org*

The prevalence of VRE was computed by region, type of antimicrobial testing (AST) method, study period, types of specimen used to isolate enterococci, and publication history. The prevalence of VRE by region was 26.1% (95% CI: 10.7–50.9%; *I*^*2*^ *=* 41.65%; *P* = 0.113) in Addis Ababa, 15.0% (95% CI: 6.9–29.6%; *I*^*2*^ *=* 79.39%; *P* <  0.001) in Amhara, 9.0% (95% CI: 2.8–25.7%; *I*^*2*^ = 71.49%; *P* = 0.015) in Oromia and 1.9% (95% CI: 0.1–23.1%) in Southern nations, nationalities and peoples region (SNNPR) (Table 3, Fig. 4). The prevalence of VRE pooled from studies conducted in the period before 2015 was 16.5% and that of the post-2015 was 16.3%; which indicates unchanged trend of VRE infections in Ethiopia. On the other hand, the pooled prevalence of VRE from studies which used disc diffusion to determine AST was 16.9% and it was 7.9% when AST was measured by dilution/minimum inhibitory concentrations (MICs). Relatively; high rates of VRE were isolated from urine (37.3%) and blood (22.0%) specimens. Use of multisite specimens did not increase the isolation rate of *enterococci*. Unpublished studies reported high rate of VRE than published studies (31.9% Vs. 11.3%; respectively) (Table 3).

**Table 3:** Pooled prevalence of VRE by subgroups

Subgroups	Numbers of studies	No of enterococci isolates tested, N	Pooled prevalence of VRE, N (%)	95% CI	*I*^*2*^	*P-value*
Region						
Addis Ababa	7	56	13 (26.1)	10.7–50.9	41.65	0.113
Amhara	8	568	38 (15.0)	6.9–29.6	79.39	< 0.001
Oromia	4	154	19 (9.0)	2.8–25.7	71.49	0. 015
SNNPR	1	53	1 (1.9)	0.8–19.8	–	–
Study period^a^						
Before/in 2015	8	354	27 (16.5)	6.5–31.5	81.09	< 0.001
After 2015	11	424	43 (16.3)	7.6–31.3	69.20	< 0.001
AST method						
Disc diffusion	17	649	61 (16.9)	9.3–28.9	66.89	< 0.001
Dilution/MIC	3	182	10 (7.9)	1.9–27.6	91.88	< 0.001
Type of specimen						
Stool	5	598	37 (5.9)	2.8–11.7	0.00	0.629
Urine	4	26	10 (37.3)	15.8–63.3	0.00	0.665
Blood	4	26	4 (22.0)	6.9–51.9	45.06	0.141
Wound swab	1	2	1 (50.0)	–	–	–
Multi-site^b^	6	179	19 (16.8)	8.0–31.9	77.87	< 0.001
Publication history						
Published	14	792	60 (11.3)	6.4–19.2	72.86	< 0.001
Unpublished	6	39	11 (31.9)	12.9–59.7	25.82	0.241

^a^ One study did not report its study period; ^b^ Studies used more than one type of specimen to isolate enterococci; MIC: Minimum Inhibitory Concentration

### Antimicrobial resistant *enterococci*

The resistance profile of *enterococci* was also pooled for antimicrobials other than Vancomycin. Resistance rates were pooled if at least two studies reported on a specific bacterium-antibiotic combinations. High level of resistance was observed to all classes of tested antimicrobials except to Daptomycin and Linezolid. The pooled resistance rate of enterococci to Daptomycin was 3.2% (95% CI; 0.5–19.7%) and that of Linezolid was 9.9% (95% CI; 2.8–29.0%). The pooled resistance rate to other antimicrobials was 60.7% (95% CI; 39.2–78.3%) to Penicillin, 56.5% (95% CI; 49.6–63.2%) to Amoxicillin, 53.7% (95% CI; 35.8–70.7%) to Tetracycline, 55.1% (95% CI; 22.2–84.9%) to Doxycycline, and 49.6% (95% CI; 36.5–62.7%) to Erythromycin. Studies reporting resistance to three or more antimicrobials were also pooled to estimate the prevalence of multidrug resistant (MDR) *enterococci* in Ethiopia. Hence; the overall prevalence of MDR *enterococci* was 63.0% (95% CI; 48.6–75.4%; I^2^ = 90.27%; *P* <  0.001) (Table 4).

**Table 4:** Pooled resistance profile of *enterococcal* isolates in Ethiopia

Antibiotics	No of studies	No of enterococci isolates tested, N	Pooled resistance N, (%)	95% CI	*I*^*2*^ *(%)*	*P-value*
Amoxicillin	2	203	115 (56.5)	49.6–63.2	0.00	0.382
Amox-clavulanate	2	225	71 (45.3)	13.9–80.9	92.37	< 0.001
Ampicillin	16	807	344 (44.5)	29.2–61.0	90.83	< 0.001
Chloramphenicol	12	777	188 (32.9)	20.8–47.8	87.24	< 0.001
Ceftriaxone	2	8	4 (50.0)	20.0–80.0	0.00	> 0.05
Ciprofloxacin	17	765	266 (36.5)	27.0–47.3	75.30	< 0.001
Clindamycin	4	224	59 (26.9)	21.5–33.2	0.00	0.478
Daptomycin	2	29	0.5 (3.2)^a^	0.5–19.7	0.00	0.974
Doxycycline	3	254	85 (55.1)	22.2–84.0	90.21	< 0.001
Erythromycin	14	780	374 (49.6)	36.5–62.7	86.19	< 0.001
Gentamycin	10	533	248 (37.7)	22.2–56.1	88.86	< 0.001
Linezolid	2	30	2 (9.9)	2.8–29.0	0.00	0.336
Nitrofurantoin	9	404	117 (31.5)	23.4–41.0	38.76	0.110
Norfloxacin	5	350	100 (39.9)	18.6–66.6	90.21	< 0.001
Penicillin	8	343	181 (60.7)	39.9–78.3	86.63	< 0.001
Streptomycin	3	179	74 (36.8)	10.4–73.1	91.62	< 0.001
Tetracycline	9	450	199 (53.7)	35.8–70.7	86.95	< 0.001
SXT	10	241	104 (39.1)	21.48–59.6	45.58	0.088
MDR - enterococci	20	825	543 (60.0)	42.9–75.0	90.27	< 0.001

*SXT* Trimethoprim-Sulfamethoxazole, *MDR* Multidrug resistance

^a^ Continuity correction (0.5) is added to the study

## Discussion

Determining the prevalence of antibiotic resistance is an important step in the formulation of interventions to control emergence and transmission of resistant pathogens. In recent years, an increase in invasive VRE infections have been reported elsewhere in the worldwide [13, 17, 25, 49]. Although antimicrobial resistance surveillance centers were established and priority surveillance pathogens were identified to prevent the spread of drug resistance in Ethiopia, VRE were not included in the lists of priority pathogens. A previous systematic review [26] reporting the growing challenges of antibacterial resistance in Ethiopia had not assessed the burden of drug resistant *enterococci*. The prevalence of VRE has been reported by several studies in Ethiopia but a comprehensive review covering different parts of Ethiopia has not been conducted. This systematic review and meta-analysis was conducted to estimate the pooled prevalence of VRE and antimicrobial resistance profile of *enterococci* in Ethiopia.

Twenty studies reporting the prevalence and/or number of VRE isolates were included in this study. Majority (80%) of the included studies failed to report the isolated *enterococci* at species level and simply highlighted the corresponding antimicrobial resistance profile. This might be due to poor laboratory capacity to identify species of *enterococci.* This indirectly indicates the potential existence of drug resistant *enterococci* in health care settings in Ethiopia and possible spread to the communities unless appropriately maintained., Although there was considerable methodological difference between studies, they were pooled for the purpose of this review. Therefore; the pooled prevalence of VRE in Ethiopia was estimated at 14.8%. This estimate is comparable with reports from Iran (14, 18.75%) [50, 51].

On the other hand, our finding was lower than studies reported from North America (21%), Asia (24%) and Europe (20%) [52]. Another study from Iran reported high rate of VRE (48.9%) among hospitalized patients [53]. These differences might be related with study population that hospitalized and critically ill patients are more likely to acquire VRE [13, 54] than the largely non-hospitalized study populations pooled in our analysis. In addition, the study period may contribute for the high rate of isolation in these countries. The studies were also conducted in the 1990’s and 2000’s following the first reports of VRE [21, 22]; while all of the studies included in our analysis were done in the 2010’s where clinical use of Vancomycin was being discouraged [11].

In contrast, higher rates of VRE was observed in our study than reports from Singapore (9.3%) [55], Germany (9.8%) [49], Iran (9.4%) [56] and United Kingdom (9.2%) [57]. Different factors were identified as risk factors for acquiring VRE infections including previous hospitalization, patient transfer, urinary catheters, critical illnesses, underlying diseases, contact with VRE patients and inappropriate use of antibiotics [54, 55, 58, 59]; all of which could contribute for the high prevalence of VRE in Ethiopia. Generally, infections and colonization with VRE were reported to be associated with health care contacts [18]. This could be true in settings where infection control knowledge, attitudes and practices among healthcare workers is poor in Ethiopia [60]. High frequency of inappropriate use of antibiotics and empirical therapies by healthcare professionals was also reported in Eastern Ethiopia [61]. In addition, the antimicrobial susceptibility testing method was based chiefly on disc diffusion and resistance was defined following the CLSI guideline.

Regional prevalence of VRE was also estimated. The highest estimated prevalence was obtained from Addis Ababa (26.1%); almost two times higher than Amhara (15.0%) and three times higher than Oromia (9.0%). This regional difference might be attributed by different study settings (hospital set up), study period, study population, variation in antibiotic use, method of antimicrobial susceptibility testing and type of specimens used to isolate *enterococci*. Stool, urine and blood were the most common specimens from which VRE were isolated. This is not surprising because *enterococci* have been reported as the most common organisms isolated from intestinal tract, urinary tract and blood stream infections [5, 8, 15, 20, 48, 52, 57, 62].

*Enterococci* are not only resistant to Vancomycin but also to other commonly used antimicrobials including Penicillin, Amoxicillin, Doxycycline, Tetracycline, Erythromycin, Daptomycin, Linezolid and others (see Table 4 above). Multidrug resistant (MDR) enterococci were also observed that could pose a critical health problem in patients and health care settings in Ethiopia. As there is no specific recommendation for the antimicrobial prescription of VRE and a follow up surveillance is not conducted at different health care centers where the studies included in this review were conducted, the prevalence of VRE is expected to continuously increase. With these concerns in mind, there has been success stories in treating VRE infections with Daptomycin and Linezolid [62]. In our analysis however; resistance to Daptomycin and Linezolid was observed in about 3.2 and 9.9% of enterococcal isolates, respectively. Although it requires strong studies, our analysis indicated that these drugs may select vancomycin resistant strains in some potentially pathogenic *enterococci* through antibiotic selection pressure as they showed some sort of resistance to Daptomycin and Linezolid.

### Strengths and limitations of the study

A comprehensive search with clear inclusion and exclusion criteria was used, examined commonly used specimens and methods of susceptibility testing, and included unpublished studies retrieved from Addis Ababa University repository. The Trim-and-Fill method was applied to asymmetric funnel plots to produce adjusted estimates. There were a number of limitations in the depth and breadth of data. ***First***; inability to report pooled estimates of VRE at species level due to the paucity of included studies reporting *enterococci* at species level. ***Second***; the definition of VRE was not consistent across studies and different AST methods were combined limiting comparability and strength of this analysis. ***Third;*** data was not available from 54.5% of the regions, outside health care setting and non-human studies were excluded that may be difficult to generalize the pooled results. ***Fourth;*** combing resistance results from different patients across different regions might pool out the peaks of resistance in some settings. ***Lastly;*** the study protocol was not registered at PROSPERO.

## Conclusion

The prevalence of VRE and drug resistant *enterococci* are on the rise in Ethiopia. Enterococcal isolates showed resistance to one or more of the commonly prescribed drugs in different or the same drug lines. Multidrug resistant (MDR) *enterococci* were also found. Although the rates were low, the emergence of resistance to Daptomycin and Linezolid is an alarm for searching new ways for the treatment and control of VRE infections. This review provides data about the current burden of VRE in Ethiopia and showed gaps that would be addressed in future studies to maintain the spread of VRE infections. Adherence to antimicrobial stewardship, comprehensive testing and ongoing monitoring of VRE infections in the health care settings are required.

## Supplementary information

**Additional file 1.** The Joanna Briggs Institute (JBI) Critical Appraisal Checklist for Studies Reporting Prevalence Data

**Additional file 2.** Quality of the included 20 studies evaluated by JBI critical appraisal checklists

## Data Availability

The datasets used and/or analyzed during the current study are included in the manuscript.

## Abbreviations

AMR: Antimicrobial resistance
ART: Antiretroviral Therapy
AST: Antimicrobial Susceptibility Testing
CLSI: Clinical and Laboratory Standards Institute
EPHI: Ethiopian Public Health Institute
JBI: Joanna Biggs Institution
MDR: Multi Drug resistance
MIC: Minimum Inhibitory Concentration
SXT: Trimethoprim-Sulfamethoxazole
VRE: Vancomycin Resistant Enterococci
WHO: World Health Organization
