# Supplementary material for: Prevalence of Vancomycin resistant enterococci (VRE) in Ethiopia: a systematic review and meta-analysis
Source: BMC Infect Dis. 2020 Feb 11;20:124. doi: 10.1186/s12879-020-4833-2 (PMC7014939; doi:10.1186/s12879-020-4833-2)
Supplement: Supplementary file 2 — Additional file 2. Quality of the included 20 studies evaluated by JBI critical appraisal checklists [file 12879_2020_4833_MOESM2_ESM.docx]

**Quality of included studies by JBI critical appraisal checklist for studies reporting prevalence data**

| Study name | **Checklists*** | | | | | | | | | **Overall** |
| --- | --- | --- | --- | --- | --- | --- | --- | --- | --- | --- |
|  | **1** | **2** | **3** | **4** | **5** | **6** | **7** | **8** | **9** |  |
| 1. Abamecha, 2015 | Yes | **No** | Yes | Yes | Yes | Yes | Yes | Yes | Unclear | 7 |
| 1. Abebe, 2014 | Yes | Yes | Yes | Yes | Yes | Yes | Yes | Yes | Yes | 9 |
| 1. Agegne, 2018 | Yes | Yes | Yes | Yes | Yes | Yes | Yes | Yes | Yes | 9 |
| 1. Ali, 2018 | Yes | Yes | Yes | Yes | Yes | Yes | Yes | Yes | Yes | 9 |
| 1. Ayelign, 2018 | Yes | Unclear | Yes | Yes | Yes | Yes | Yes | Yes | Yes | 8 |
| 1. Birri, 2013 | Yes | **No** | **No** | Yes | Yes | Yes | Yes | Yes | Yes | 7 |
| 1. Eshetu, 2017 | Yes | Yes | Yes | Yes | Yes | Yes | Yes | Yes | Yes | 9 |
| 1. Fentie, 2018 | Yes | Yes | Yes | Yes | Yes | Yes | Yes | Yes | Yes | 9 |
| 1. Ferede, 2018 | Yes | Yes | Yes | Yes | Yes | Yes | Yes | Yes | Yes | 9 |
| 1. Gebrish, 2019 | Yes | Yes | **No** | Yes | Yes | Yes | Yes | Yes | Yes | 9 |
| 1. Jemal, 2017 | Yes | Yes | **No** | Yes | Yes | Yes | Yes | Yes | Yes | 8 |
| 1. Lega, 2015 | Yes | **No** | Yes | Yes | Yes | Yes | Yes | Yes | Yes | 8 |
| 1. Mitiku, 2018 | Yes | **No** | Yes | Yes | Yes | Yes | Yes | Yes | Yes | 8 |
| 1. Mohammed, 2017 | Yes | Yes | Unclear | Yes | Yes | Yes | Yes | Yes | Yes | 8 |
| 1. Molalign, 2016 | Yes | Yes | Yes | Yes | Yes | Yes | Yes | Yes | Yes | 9 |
| 1. Sorsa, 2019 | Yes | Yes | Yes | Yes | Yes | Yes | Yes | Yes | Yes | 9 |
| 1. Teklehaymanot, 2016 | Yes | Yes | Yes | Yes | Yes | Yes | Yes | Yes | Yes | 9 |
| 1. Toru, 2018 | Yes | **No** | Unclear | Yes | Yes | Yes | Yes | Yes | Yes | 7 |
| 1. Woldemariam, 2019 | Yes | **No** | Unclear | Yes | Yes | Yes | Yes | Yes | Yes | 7 |
| 1. Yilema, 2017 | Yes | Yes | Yes | Yes | Yes | Yes | Yes | Yes | Yes | 9 |

*** 1.** Appropriate sampling frame to address target population, **2.** Appropriate sampling way of study participants, **3.** Adequate sample size, **4.** Detail description of study participants and settings, **5.** Data analysis with sufficient coverage of identified sample, **6.** Use of valid methods to identify the condition, **7.** Standard, reliable way of measurement of condition for all participants, **8.** Availability of appropriate statistical analysis, **9.** Adequate response rate and management of low response rate
